# Ligand binding to an Allergenic Lipid Transfer Protein Enhances Conformational Flexibility resulting in an Increase in Susceptibility to Gastroduodenal Proteolysis

**DOI:** 10.1038/srep30279

**Published:** 2016-07-26

**Authors:** Syed Umer Abdullah, Yuri Alexeev, Philip E. Johnson, Neil M. Rigby, Alan R. Mackie, Balvinder Dhaliwal, E. N. Clare Mills

**Affiliations:** 1 Institute of Food Research, Norwich Research Park, Colney, NR4 7UA, UK; 2 Institute of Inflammation and Repair, Manchester Academic Health Sciences Centre and Manchester Institute of Biotechnology, University of Manchester, 131 Princess Street, Manchester, M1 7DN, UK

## Abstract

Non-specific lipid transfer proteins (LTPs) are a family of lipid-binding molecules that are widely distributed across flowering plant species, many of which have been identified as allergens. They are highly resistant to simulated gastroduodenal proteolysis, a property that may play a role in determining their allergenicity and it has been suggested that lipid binding may further increase stability to proteolysis. It is demonstrated that LTPs from wheat and peach bind a range of lipids in a variety of conditions, including those found in the gastroduodenal tract. Both LTPs are initially cleaved during gastroduodenal proteolysis at three major sites between residues 39–40, 56–57 and 79–80, with wheat LTP being more resistant to cleavage than its peach ortholog. The susceptibility of wheat LTP to proteolyic cleavage increases significantly upon lipid binding. This enhanced digestibility is likely to be due to the displacement of Tyr79 and surrounding residues from the internal hydrophobic cavity upon ligand binding to the solvent exposed exterior of the LTP, facilitating proteolysis. Such knowledge contributes to our understanding as to how resistance to digestion can be used in allergenicity risk assessment of novel food proteins, including GMOs.

The non-specific lipid transfer proteins (nsLTPs) are a group of plant proteins initially defined by their ability to transfer phospholipids from liposomes to mitochondria *in vitro*, in a non-specific manner with regards to both the type of phospholipid and membrane^1^. They are widely found in plant tissues, with >100 proteins annotated as nsLTPs or putative nsLTPs in the model plant species *Arabidopsis thalinia* alone^2^ indicative of their diverse biological roles in plants. The first allergenic LTP from peach was identified more than a decade ago^3^,^4^, since when LTPs have been found to be the major allergens in many foods, leading to the family being termed as ‘pan-allergens’^4^. Allergies to LTPs are generally found in populations living around the Mediterranean area^5^ where it is an important type of food allergen accounting for sensitization in more than 90% of patients allergic to peach alone in this region of Europe, and is associated with severe, life-threatening reactions including anaphylaxis. More recently it has emerged that LTPs may be important for allergies to fruits such as peach in Northern Europe^6^ and have been implicated as important allergen molecules in severe forms of hazelnut allergy^7^. Similarly, wheat LTP is a major allergen associated with baker’s asthma^8^-an occupational asthma found in bakery employees, and food allergy^9^.

LTPs are small, ~9 kDa proteins comprising a bundle of four α-helices packed against a C-terminal region and belong to the prolamin superfamily of allergens^10^. Eight conserved cysteines are characteristic of the superfamily, notably the Cys-Cys and Cys-X-Cys motifs, (where X represents any other residue). These cysteines form four intra-chain disulphide bonds configured to create a hydrophobic tunnel capable of binding a variety of lipophilic molecules. The structures of several free and liganded LTPs have been determined, including those from barley, wheat and peach^11^,^12^,^13^ and a post-translationally modified form of barley LTP1, LTP1b, in which a lipid-like adduct is attached to the protein via the side chain of Asp 7^14^,^15^. Molecular dynamics studies have indicated that the hydrophobic lipid binding tunnel of nsLTPs is plastic in nature^16^, observations supported by the fact the cavity expands from 250 Å^3^ to 750 Å^3^ on binding di-myristoyl-phosphatidyl-glycerol^17^ and that the adducted LTP1b of barley has increased flexibility^18^.

It has been proposed that resistance to digestion may play an important role in determining the ability of certain proteins to sensitise naïve individuals and that factors such as stability and solubility may facilitate transfer of allergen into the circulation and hence potentiate severe allergic reactions^19^. As a consequence resistance to pepsin digestion is used as part of the allergenicity risk assessment of genetically modified organisms (GMOs)^20^. We have previously shown that the resistance of LTPs to gastric proteolysis is a result of the structural stability of this protein to the low pH conditions of the stomach^21^. However, amino acid side chain mobility may play an important role in determining susceptibility to hydrolysis by intestinal proteases trypsin and chymotrypsin, with the increased susceptibility of the lipid adducted LTP1b suggesting ligand occupancy might enhance digestion by increasing polypeptide mobility^21^. We have now tested this hypothesis by investigating the effect of ligand binding on the susceptibility of peach and wheat LTPs to simulated gastroduodenal digestion, using the widely found plant lipid linoleic acid.

## Results

### Wheat and peach LTP ligand binding studies

**Figure 1:**
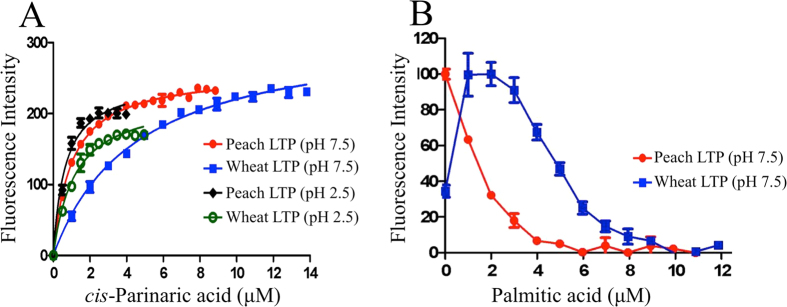
Ligand binding to peach and wheat LTP. (**A**) CPA binding to peach or wheat LTPs at pH 2.5 and 7.5. (**B**) Example CPA displacement assays (using palmitic acid as the competitive ligand) highlighting the difference in the wheat and peach LTP fluorescence curves. Each data point is the mean of three replicate experiments, with the standard deviation error shown.

The ligand binding activities of peach and wheat LTP were compared using *cis*-parinaric acid (CPA) in a fluorescence-based assay^22^: CPA is a naturally occurring polyunsaturated fatty acid with intrinsic fluorescent properties, the quantum yield of which is sensitive to changes in solvent. CPA bound to peach LTP with greater affinity than to wheat LTP (Fig. 1A), with *K*_d_ values of 1 and 4.5 μM, respectively (Table 1). A CPA displacement assay was then used order to study the binding of other commonly occurring ligands which are non-fluorescent. Since palmitic acid and linoleic acid are the major fatty acids found in peach^23^ and wheat^24^ respectively, their binding was assessed, together with biosurfactants commonly found in the gastrointestinal tract^25^ (Table 1). The displacement curves for peach and wheat LTP differed (Fig. 1B), the latter showing an increase in fluorescence as the displacing ligand was titrated in, which then gradually decreased. Wheat LTP has previously been shown to bind multiple lipids and the bell-shaped curve could be explained by a lipid binding in the cavity alongside the CPA, which may result in more effective exclusion of solvent from the pocket, increasing the fluorescence of the CPA^11^,^26^,^27^,^28^. In general, the ligands displaced CPA much more readily from peach compared with wheat LTP, giving apparent *K*_i_ values of 0.7–1.1 μM compared to 3.6–5 μM for wheat. Multiple occupancy may also account for the lower apparent affinity of the wheat LTP for ligands, such as linoleic acid. Interestingly, both LTPs showed weaker binding to monomeric PC compared to its miceller form, which may reflect the complex interactions of LTP with vesicular lipid structures^29^. At pH 2.5 in the presence of 150 mM NaCl, a model of the pH and ionic strength conditions found in the stomach^21^, the affinity for CPA and PC increased for both proteins (Table 1).

### Mass spectrometric analysis of LTP digestion reveals three major cleavage sites

**Figure 2:**
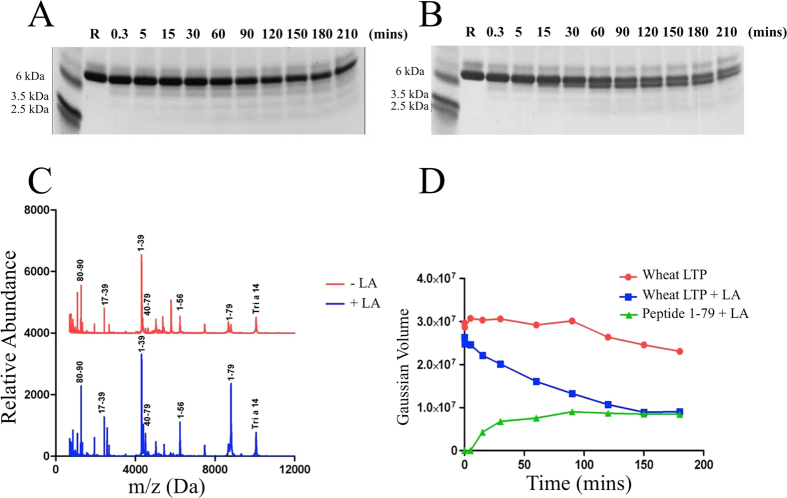
Duodenal digestion of wheat LTP. (**A,B**) SDS PAGE analysis of digestion under reducing conditions at various time points in the absence (**A**) or presence (**B**) of 0.26 mM linoleic acid (LA); lane R is a reference showing wheat LTP following the duodenal digestion procedure but in the absence of trypsin and chymotrypsin. (**C**) MALDI-ToF MS spectra of the duodenal digests of wheat LTP after 120 min in the absence (red spectra) or presence (blue) of 0.26 mM linoleic acid. The peptides were relativity quantified by comparing spectral intensities of the *same* peptide in the absence or presence of linoleic acid. (**D**) Densitometric analysis of SDS PAGE shown in (**A**,**B**).

The effect of lipid binding on the stability of wheat and peach LTPs to gastroduodenal digestion was assessed using linoleic acid as a representative ligand. Both LTPs were resistant to pepsinolysis in both liganded and unliganded forms, as judged by SDS-PAGE and MALDI-ToF MS (Figures S1 and S2), as previously described^21^,^30^. However, following *in vitro* gastric digestion, they were digested albeit to a limited extent, by the duodenal proteases trypsin and chymotrypsin (Figs 2 and S3): Mass spectrometry profiling using MALDI-ToF under reducing conditions confirmed previous observations that peach LTP is digested to yield a large 8334.09 Da fragment corresponding to residues 1–79 which is further degraded at later stages of digestion into two fragments corresponding to residues 1–39 and 40–79 (4200.8 Da) (Figure S3)^21^. The wheat LTP, like the closely homologous LTP from barley, was somewhat more resistant to gastroduodenal proteolysis with 80% of the protein remaining intact after 120 min, as assessed by densitometric analysis of SDS-PAGE gels (Fig. 2D).

**Figure 3:**
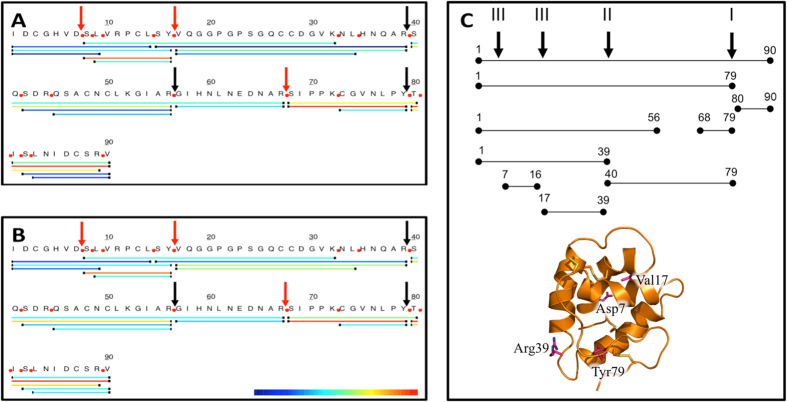
Mapping of *in vitro* gastroduodenal digestion products of wheat LTP using MS/MS with either LTP alone (**A**) or in the presence of 0.26 mM linoleic acid (**B**). Digestion products were mapped on the primary sequence of wheat LTP (Uniprot ID P24296) (**A,B**), coded by relative intensity with blue indicating the lowest and red indicating the highest intensity. Arrows mark the major experimentally determined cleavage sites. Digestion products were mapped onto a cartoon representation of wheat LTP (**C**), with the major cleavage sites displayed.

MALDI-ToF spectra of reduced wheat LTP after 120 minutes of duodenal digestion showed the presence of 23 mass events of which 16 could be assigned as wheat LTP fragments based on accurate parent ion mass (Fig. 3A and Table 2). In addition to the intact protein, major peptide fragments identified corresponded to residues 1–39, 1–56, 1–79, 17–39 and 40–79, with a number of other lower intensity peptides also being identified. Digestion products were also characterised by MS/MS (Fig. 3) to provide unambiguous identification of peptides although it is applicable only to low mass ranges (typically below 3500 Da in our experience). These data confirm the major cleavage sites observed by MALDI-ToF MS and identified several smaller peptides, presumably derivatives of the high molecular weight peptides corresponding to residues 1–79, 1–39, 40–79 and 1–56. Normalised relative intensity allowed the relative abundance of the peptides to be mapped on to the wheat LTP sequence (Fig. 3A) and shows that the major peptide products detected corresponded to residues 9–16, 68–79 and 80–90. Combining the MALDI-ToF and MS-MS mapping of the major proteolysis products shows the presence of four major cleavage events. (Figure 3C) corresponding to three chymotryptic sites, Asp7-Ser8, Tyr16-Val17 and Tyr79-Thr80 together with a single tryptic site corresponding to Arg39-Ser40. Mapping of the major digestion products onto the LTP structure showed these sites reside in loop regions on the surface of the protein (Fig. 3C).

### Wheat LTP digestibility is increased upon lipid binding

Loading peach LTP with linoleic acid did not affect the rate of digestion (Figure S3) whereas lipid loading of wheat LTP increased its digestibility such that only around 30% of the protein remained intact after 120 min digestion (Fig. 2), with a lower mass (~8.5 kDa) stable digestion product evident on SDS-PAGE. This polypeptide has a mass similar to the abundant peptide identified by MALDI-ToF MS of 8803.44 Da corresponding to residues 1–79. This peptide is ~8-fold more abundant in the MALDI-ToF spectrum for wheat LTP digested in the presence of lipid (Fig. 2C). The abundance of many other assigned peptides were also slightly higher during digestion in the presence of linoleic acid. The pattern of proteolytic digestion products mapped by MS/MS analysis (Fig. 3B) was identical, to that obtained in the absence of lipid, indicating that the lipid occupancy simply enhanced the rate of proteolysis and did not affect the pattern of peptide products.

### Tyr79 is displaced upon ligand binding to wheat LTP

Ligand binding has been shown to increase side-chain mobility within the LTP cavity^18^. Side-chain dynamics have in turn been shown to be an important factor in determining the cleavability of LTP by trypsin and chymotrypsin^21^. Therefore, differences in side-chain dynamics may explain why loading wheat LTP with ligand increases its susceptibility to gastroduodenal proteolysis. A comparative structural analysis of wheat and peach LTPs was conducted by superposing the structures of unliganded wheat LTP (PDB 1GH1), wheat LTP bound to either a single molecule of prostaglandin B_2_ (1CZ2) or two phospholipid molecules (1BWO), and peach LTP bound to heptane and/or lauric acid (2ALG). Overall the structures are similar (RMSD range of 1.5–1.8 Å for all Cα atom pairs), with the unstructured C-terminal region (residues 74–90) expectedly showing the greatest variation (RMSD of 3.1–4.0 Å) (Table S1).

**Figure 4:**
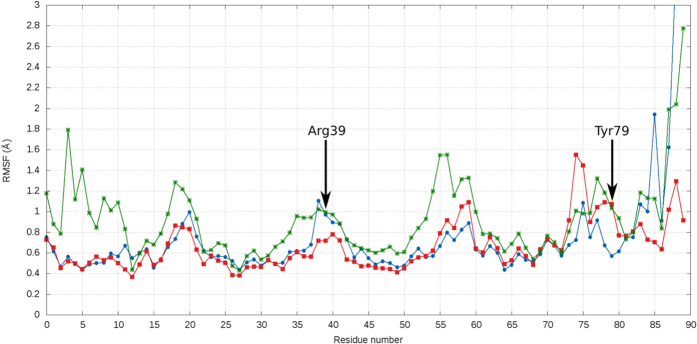
RMS fluctuations of unliganded and liganded wheat LTP side-chains. The blue, green and red lines show RMSF values for unliganded (based on PDB 1GH1), one linoleic acid molecule bound (1CZ2) and two linoleic acid molecules bound (1BWO) wheat LTPs, respectively. (The C-terminal residue Val90 of the unliganded wheat LTP is extremely flexible and has a RMSF value of 5.9 Å). The key proteolytic cleavage site residues 39 and 79 are labelled.

How does lipid binding to wheat LTP displace Tyr79 from the central hydrophobic cavity? Molecular dynamics (MD) simulations of peach LTP had previously been conducted to analyse lipid binding and the plasticity of the central cavity^13^. Intriguingly the authors noted the side-chain of Tyr79 as being particularly flexible. Therefore we conducted MD simulations on wheat LTP. The global conformational flexibility of the protein is found to vary significantly upon the binding of one or two ligands, with the overall average residue RMS fluctuation (RMSF) values for each wheat LTP protein complex (0.8 Å, 0.9 Å and 0.7 Å for unliganded, one- or two-linoleic lipids bound wheat LTPs, respectively) revealing that wheat LTP bound to one ligand is structurally *less* stable than when it is bound to two ligands or is unliganded (Fig. 4). This is an unexpected finding as proteins are often stabilized upon binding of a ligand. Furthermore, the conformational flexibility of wheat LTP can be divided into two distinct regions: The N-terminal region is more flexible when the protein is bound to one ligand, compared to the unliganded or two ligands-bound forms. The C-terminal tail of wheat LTP is very flexible when unliganded or bound to one ligand, but stable when two ligands are bound. Interestingly, a subregion comprising Tyr79 and proximal residues is the only moiety where flexibility of the unliganded wheat LTP is lower than that of its liganded forms. Specifically, the RMSF of Tyr79 increases from 0.7 Å in the unliganded form of wheat LTP, to 1.2 Å and 1.0 Å when one or two ligands are bound respectively (Fig. 4). Therefore, taken together with the experimentally determined structures of wheat LTP, MD simulations show that upon ligand binding the flexibility of Tyr79 and its neighboring residues are increased, allowing the residues to adopt positions outside the hydrophobic cavity. The solvent accessible residues are thus more susceptible to proteolytic cleavage. The increased flexibility of side-chains participating in proteolysis has previously been linked to the increased digestibility of a protein^31^ and is consistent with the presented experimental results.

## Discussion

The mass-spectrometric profiling and SDS-PAGE analysis of the *in vitro* gastroduodenal digestion of wheat LTP show that whilst the pattern of proteolytic digestion products remain unaffected, the *rate* of digestion is increased significantly in the presence of a lipid ligand, such that its digestibility more closely resembled that of peach LTP.

**Figure 5:**
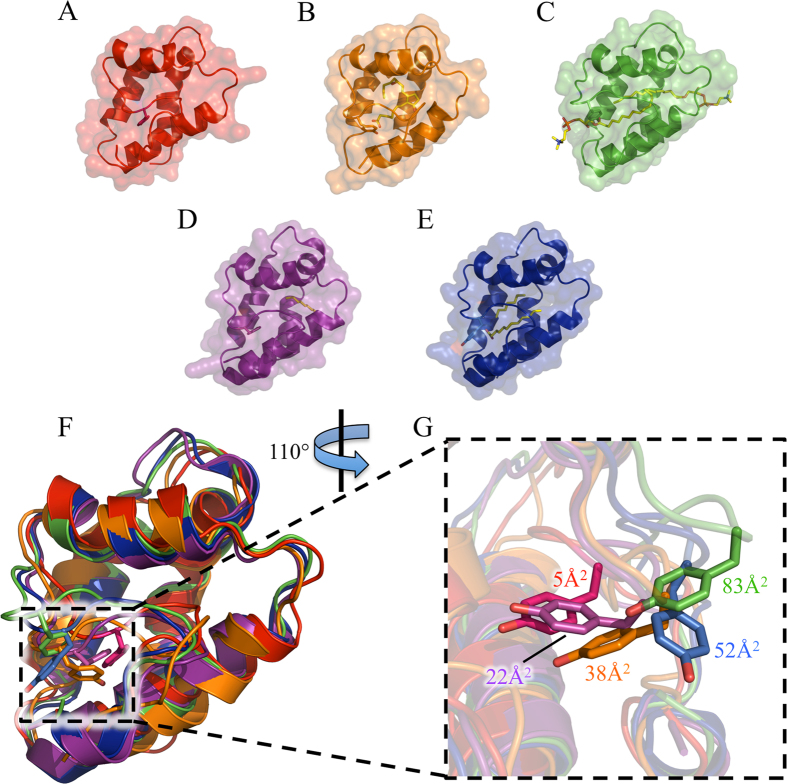
Structural comparison of wheat and peach LTPs. (**A–E**) Structures of wheat and peach LTPs (shown as cartoon Cα traces with transparent van der Waals surfaces. Ligands and Tyr79 side-chains are shown in all-atom stick representation): (**A**) Unliganded wheat LTP (PDB 1GH1, ensemble model 11)–colored red; (**B**) Prostaglandin B_2_-bound wheat LTP (1CZ2, ensemble model 10)–orange; (**C**) wheat LTP bound to two molecules of phospholipid (1BWO, molecule A)–green; (**D**) peach LTP bound to a lauric acid molecule partially occupying the central cavity^13^ (2ALG, molecule B)–purple; (**E**) peach LTP bound to lauric acid and heptane (2ALG, molecule A)–blue. (**F**) Superposition of the wheat and peach LTP structures, Tyr79 are shown. (**G**) Enlarged view highlighting the positions of Tyr79 in the wheat and peach unliganded and ligand-bound structures. Also shown are the solvent accessible surface areas for each Tyr79 residue (see Table 3).

Examination of the two main sites for proteolytic cleavage of wheat LTP by trypsin and chymotrypsin, Arg39 and Tyr79^21^ respectively, revealed that whilst the orientation of the Arg39 side-chain remains essentially unchanged, Tyr79 is conformationally dynamic: In the unliganded form of wheat LTP, Tyr79 has a solvent accessible surface area (SASA) area of just 5 Å^2^ (Table 3 and Fig. 5), indicating that the residue is almost inaccessible to solvent. Upon the binding of a single lipid molecule by wheat LTP, the SASA of the interacting Tyr79 residue increases 7-fold to 38 Å^2^. The binding of a second ligand results in the solvent exposure of Tyr79 further increasing to 83 Å^2^. Turning to peach LTP, its crystal structure has two molecules present within the asymmetric unit^13^, with molecule B binding a single lauric acid molecule in an unusual manner (Fig. 5D). The lipid only partially occupies the central cavity and is distant from Tyr79 (9 Å). However, Tyr79 of this molecule is found to be partly solvent accessible (22 Å^2^). Molecule A of the peach LTP structure has both heptane and lauric acid bound within the central cavity in a conventional mode, and the molecule’s Tyr79 residue is exposed to solvent (52 Å^2^) (Fig. 5). The wheat and peach LTP structural data are consistent with our results, namely that the chymotryptic proteolysis of peach LTP is unaffected by the presence of lipid likely due to the Tyr79-Thr80 cleavage site being solvent exposed in both “unliganded” and liganded forms. The rate of wheat LTP proteolysis is enhanced upon the addition of lipid most probably because Tyr79 is displaced from an almost inaccessible cavity to a solvent exposed environment.

Molecular dynamic simulations, coupled with structural and mass spectrometric data, suggest a mechanism for the enhanced digestibility of wheat LTP in the presence of ligand. In addition to the well-characterized expansion of the hydrophobic cavity upon lipid binding, the side-chains of Tyr79 and neighbouring residues increase in flexibility, allowing Tyr79 to flip out of the hydrophobic pocket to the solvent exposed exterior of LTP, residues 79–80 consequently become more susceptible to chymotryptic cleavage. Subsequently, it appears that the LTP is cleaved at residues 39–40 by trypsin, a slower event since the pH in the *in vitro* digestion model is below the optimum for trypsin.

The interaction of LTPs and lipids may not only modulate the immune system by varying the susceptibility of these potentially allergenic proteins to digestion, but the interaction may also be important in the defence against antimicrobial infection (speculated to result from LTPs interacting and permeabilizing biological membranes^32^); the presentation of lipid antigens to T-cells^33^; and allergen uptake by intestinal epithelial cells^34^. Therefore differences in lipid binding affinities may reflect differences in the immunomodulatory activities of LTPs.

In most proteins ligand binding reduces the digestibility of regions of the protein in limited proteolysis experiments^35^,^36^,^37^. This is thought to be due to formation of additional hydrogen bonds between ligand and protein that stabilise the protein scaffold, increasing rigidity, and resulting in a decreased rate of proteolysis. However, in the case of wheat LTP, ligand binding increases susceptibility to proteolysis in our limited trypsin-chymotrypsin system, and runs counter to results in grape and sunflower LTPs where resistance to digestion is enhanced by the presence PC vesicles^38^,^39^. Our surprising observations suggest the response of the wheat LTP structure to ligand binding is unusual. Our findings are, however, consistent with previous work on peach and barley LTPs^14^,^18^,^21^. Barley LTP has a unique plastic hydrophobic cavity^14^ and studies with a covalent lipidic adduct suggest changes in mobility in the region of the C-terminus occur upon occupancy of the hydrophobic cavity^18^. Such mobility is consistent with B-factor analyses of LTP crystal structures that show the C-terminal region becomes more flexible on ligand binding. This is supported by our previous study of peach and barley LTP^21^, where we demonstrated that the flexibility of residues 39 and 80 are critical for proteolysis.

Bakan *et al.*^40^ suggest that changes in surface properties of LTP upon ligand binding might reflect its biological functionality. It is striking that Tyr79 is absolutely conserved in the LTPs of higher plants. These observations suggest that Tyr79 may be involved in signalling a state of ligand occupancy *via* conformational changes. This is similar to the mechanism by which mammalian fatty acid binding proteins (FABPs) appear to signal ligand occupancy *via* phosphorylation of a surface accessible tyrosine^41^. However, in the case of FABPs, the mechanism by which this tyrosine becomes available for tyrosine kinase action upon ligand binding remains unclear.

This report indicates that, in addition to their role as immune modulators, lipids could modulate the allergenicity of proteins^34^ both by modifying an allergen’s structure and physiochemical properties as well as acting directly on the immune system^42^. As members of the prolamin superfamily, plant LTPs share a structural similarity to the plant 2S albumins, for example, the lipid-binding major Brazil nut allergen Ber e 1. Recently, Mirotti *et al.*^43^ demonstrated that lipids modulate the immune responses to Brazil nut in mouse and human cell model systems. The capacity of lipids to modify the conformation of LTPs may prove to be important in understanding how such interactions affect allergenic potential and runs counter to current dogma that lipid ligands increase resistance of allergens to digestion^34^. Our observations show that structural changes in wheat LTP occur primarily at residues 79–80 upon lipid binding, enhancing its digestibility.

Further studies into the relationship between the processing of food proteins, their proteolysis and effects on allergenicity are required. Following processing, proteins may retain their native folds, or unfold (completely or partially) leading to the formation of aggregates^44^ with a modified allergenic potential^45^. After ingestion, many proteins that are susceptible to proteolysis retain their allergencity (review^46^). In the study presented here, whilst a proportion of the wheat and peach LTPs remain intact after simulated duodenal digestion, the digested protein consists of large peptide fragments, with the four-disulphide bonds disposed such that the peptide digestion products will be held together retaining much of the three-dimensional architecture of the undigested protein as we have previously demonstrated for the peach and barley homologues^21^, and thus may be capable of decreased levels of IgE binding. Studies will also be required using LTPs from different plant sources to assess further the correlation of structural dynamics, particularly of Tyr79, and stability to digestion. It maybe that lipid binding reduces Tyr79 mobility in certain LTPs, such as those from grape and sunflower and hence increases their resistance to digestion.

Such knowledge contributes to the weight of evidence approach used in the allergenicity risk assessment of novel food proteins, including newly expressed proteins in GMO food crops^20^, which takes into consideration measures of protein digestibility.

## Materials and Methods

### Protein Preparations

Wheat LTP was purified from wheat bran using a modified protocol previously described for barley LTP^47^. Briefly, the wheat bran was defatted using hexane, followed by the addition of 3% (w/v) of polyvinylpolypyrrolidone in deionized water to adsorb soluble phenolic acids. The clarified wheat extract was then loaded onto a cationic-exchange SP-Streamline column, and protein eluted with 1M NaCl. Fractions containing LTP were concentrated before loading onto a Superdex 75 prep grade gel-filtration column. LTP was then loaded onto a Poros HS-20 cation-exchange column; protein was eluted using a 0 to 0.25 M NaCl gradient. 3.0 M ammonium sulfate was then added to the pooled fractions. The suspension was spun at 1,700 g at 10 °C before loading the supernatant onto a HP 2 hydrophobic interaction column pre-equilbrated with 20 mM Tris, 2.8 M ammonium sulfate buffer. Protein was eluted using a 2.8 to 0 M ammonium sulfate gradient. Purified wheat LTP was passed down a Sephadex G15 desalting column before freeze-drying and storing the protein at −20 °C.

Peach LTP was purified from the skin of peach fruits by a combination of ammonium sulphate fractionation and cation exchange chromatography and gel filtration according to Gaier *et al.*^48^.

### Ligand binding

Ligand binding was assessed using a fluorescence assay based on *cis*-parinaric acid originally described by Cooper *et al.*^22^. Fluorescence intensity was measured at 25 °C with a LS55 Luminescence Spectrometer (Perkin Elmer, Cambridge, UK) using a 5 mm slit width for both excitation (λ = 320 nm) and emission (λ = 420 nm) and the measurement taken for no longer than 1.5–2s. CPA (3 mM in ethanol) was titrated by 1 μL injections into 1 mL of LTP solutions (5 μM in 50 mM phosphate buffer pH 7.5) in a stepwise manner. Binding curves were fitted with GraphPad Prism using the rectangular hyperbolic function of Hill’s equation. For non-fluorescent ligands, a competitive assay was developed using CPA as a tracer ligand. CPA concentrations close to the calculated *K*_d_ of each LTP^49^,^50^ (at either 1 or 0.5 μM CPA for peach LTP and 2 or 1 μM CPA for wheat LTP in 50 mM phosphate buffer pH 7.5 or pH 2.5, 150 mM NaCl, respectively) were used. After equilibrating for 2–3 min with gentle mixing, the competing non-fluorescent ligands (1 mM of palmitic acid, 16-OH palmitic acid, 12-OH stearic acid, linoleic acid or 1-palmitoyl–sn–glycerol–3–phosphatadyl choline (PC) ethanol) were titrated into the LTP solution in 1 μL aliquots. The resulting data were fitted using a sigmoidal curve-fitting logarithm in GraphPad Prism from which the concentration able to displace 50% of the CPA (IC_50_) was calculated. *K*_i_ values were calculated according to Cheng-Prusoff equation^51^.

### Simulated gastric and duodenal proteolysis

Both wheat and peach LTPs were preloaded with linoleic acid before *in vitro* gastroduodenal digestion; linoleic acid, the most abundant lipid in wheat and peach, was solubilised in 250 mM NaOH to a final concentration of 26 mM. 100 μl of the lipid solution was then slowly added to 6 ml simulated gastric fluid^52^ containing 5 mg protein (~0.1 mM LTP), therefore establishing a LTP to lipid ratio of about 1:5. The pH of the mixture was carefully maintained between 4 and 7 using 1 M NaOH or HCl, before being placed in a 37 °C shaking incubator for an hour. Proteins (0.25 mg/ml in the final digestion mix) were then incubated with pepsin at pH 2.5 to simulate gastric proteolysis. This was sequentially followed by trypsin and chymotrypsin at pH 6.5 to mimic duodenal proteolysis, as described by Moreno *et al.*^53^. The pepsin, trypsin, and bovine R-chymotrypsin enzyme activities were 3,300 U/mg of protein calculated using haemoglobin as substrate, 13,800 U/mg of protein using BAEE as substrate, and 44 U/mg of protein using BTEE as substrate, respectively. The standardised international static *in vitro* digestion protocol, developed within the COST INFOGEST (European Cooperation in Science and Technology-Improving health properties of food by knowledge sharing of the digestive process) network, includes a 120 minutes gastric phase and a 120 minutes duodenal phase^52^. However, this was modified as an earlier *in vitro* gastroduodenal study of wheat and peach LTPs revealed that both proteins are resistant to *in vitro* gastric phase after 120 minutes, and that a time point of 60 minutes is sufficient for the evaluation of *in vitro* gastric digestion of both LTPs^54^, a finding which is consistent with our earlier studies of peach LTP^21^. The progress of proteolysis was followed by SDS-PAGE analysis under reducing conditions with 50 mM dithiothreitol using a 12% Bis-Tris gel in a NuPAGE system (Invitrogen, Groningen, The Netherlands). Proteins were visualised by Coomassie brilliant Blue safe stain (Invitrogen, Paisley, UK). The molecular weight marker contained the following proteins: Insulin A chain (2,500 Da), Insulin B chain (3,500 Da), aprotinin (6,000 Da), lysozyme (14,400 Da), trypsin inhibitor (21,500 Da), carbonic anhydrase (31,000 Da), lactate dehydrogenase (36,500 Da), glutamic dehydrogenase (55,400 Da), BSA (66,300 Da), phosphorylase B (97,400 Da), β-galactosidase (116,300 Da) and myosin (200,000 Da) (Invitrogen, Groningen, The Netherlands). Preloading of the LTP proteins with a high concentration of linoleic acid in conditions favouring binding, and therefore maximizing its effects, resulted in 70% of the wheat LTP being proteolytically cleaved during digestion. Preloading the protein with lower concentrations of linoleic acid before *in vitro* digestion would therefore present challenges in the identification of peptide fragments using MALDI-ToF or SDS-PAGE.

### Mass spectrometry

Digestion products were also analysed by MALDI-ToF mass spectrometry (MS). Analysis of high molecular weight (>3,000 Da) peptides was performed by mixing each sample with a saturated sinapinic acid (Sigma-Aldrich, Dorset, UK) matrix in 30% (v/v) acetonitrile, 0.1% (v/v) trifluroacetic acid (TFA). The target plates used were polished stainless steel (Bruker Daltonics, Coventry, UK). Samples were prepared in the presence or absence of 5 mM *tris*(2-carboxyethyl)phosphine (TCEP) depending upon whether reduction was required. Sample/matrix mixture (0.5 μL) was spotted onto the MALDI target and dried in air. The MALDI-MS measurements were performed using a Bruker UltraFlex MALDI-ToF/ToF mass spectrometer (Bruker Daltonics, Coventry, UK) equipped with a pulsed N_2_ laser (λ = 337 nm, frequency 10 Hz). Whole protein spectra were recorded over the 2000–12000 m/z range in linear mode at an accelerating voltage of 25 kV by averaging of 300 individual laser shots. The concentration of each protein solution was adjusted to give peak intensities similar to that of the ubiquitin and myoglobin calibrants used. Lower molecular weight peptides were analysed by spotting 0.5 μl of each digest onto 4-hydroxy cinamic acid (HCCA) pre-spotted anchor chip (PAC) plates (Bruker Daltonics, Coventry, UK). After drying, spots were washed with 10 μl of 10 mM ammonium phosphate containing 0.1% (v/v) TFA and again allowed to dry. Mass spectra were recorded over the 700–4000 m/z range in linear mode at an accelerating voltage of 25 kV by averaging of 150 individual laser shots.

Data analysis was performed by comparison of experimentally derived peptide masses with those predicted by *in-silico* digestion of peach and wheat LTPs by trypsin and chymotrypsin using the Mmass software package^55^ with a 100 ppm tolerance.

LC-MS/MS analysis was performed using a LTQ-Orbitrap mass spectrometer and a nanoflow-HPLC system (nanoAcquity, Waters Corp.). Peptides were applied to a pre-column (Symmetry C18 5 μm beads, 180 μm × 20 mm column, Waters Corp.) connected to a 25 cm analytical column (BEH 130 C18 1.7 μm beads, 75 μm × 250 mm column, Waters Corp.). Peptides were eluted by a gradient of 5 to 40% (v/v) acetonitrile in 0.1% (v/v) formic acid from 1 to 40 min at a flow rate of 250 nL min^−1^. Mass spectra were obtained in positive ion electrospray mode. The mass range for the survey scans was *m/z* 300–2000, resolution 60,000, with *m/z* values determined by the Orbitrap FTMS stage. The FTMS fill target was 200,000 ions with a maximum fill time of 1000 ms. The resultant monoisotopic masses were accurate to better than 10 ppm. MS/MS spectra were obtained using collision induced dissociation with collision voltage 35 V with *m/z* values determined by the Linear Ion Trap stage. The MS/MS was triggered by a minimal signal of 5000 ions with a fill target of 10,000 ions and 150 ms maximum fill time with exclusion of 4 + charge states. A maximum of 4 MS/MS spectra per survey scan were obtained by defaulting to the most abundant ions, with *m/z* values determined to better than ~0.4 Da. Charge state selection was not enabled. Dynamic exclusion was set to 1 count and 60 s exclusion with an exclusion mass window of −0.5 to +1.5 Da.

Wheat and peach LTP amino acid sequences were aligned using CLUSTAL Omega^56^ and a figure generated using ESPript 3^57^.

### Molecular dynamics simulations

The structures used for simulations were unliganded wheat LTP1 NMR structure (PDB 1GH1^58^, ensemble model 11), and the wheat LTP1 structures bound to either prostaglandin B_2_ (1CZ2^59^, ensemble model 10) or 2 phospholipid molecules (1BWO^11^, chain A). The wheat LTP1 1BWO crystal structure has a dimer in the asymmetric unit, since wheat LTP1 is monomeric at neutral pH and both polypeptides are similar, chain A was chosen for simulations. The most representative NMR structures were chosen using the OLDERADO program^60^.

Linoleic acid was used in the lipid binding experiments, but the force field parameters in all-atom (version 27) CHARMM force field are not available for this particular fatty acid. However, there are parameters for oleic acid, a similar fatty acid that differs from linoleic acid by saturation of double bond 12. Therefore we used these existing parameters for generation of linoleic acid parameters by using the CHARMM-GUI^61^ and CGenFF program version 1.0.0^62^. The CH_PENALTY varied from 0 to 2.089 for linoleic acid atoms where the vast majority of atoms had a penalty score of zero, and the single added dihedral angle had a penalty of 0.6. These penalties suggest that the generated parameters are accurate and no additional validation is required. Both prostaglandin B_2_ (in 1CZ2) and phospholipid molecules (in 1BWO) were modified with a minimum number of changes to linoleic acid for simulations (all equivalent atom coordinates were kept). Since all molecules have the same or a similar length, we believe that the resultant modelled structures are reasonable. All calculations were carried out using programs VMD^63^, CHARMM (version c37b1) and NAMD (version 2.10)^64^. CHARMM force field 27 was used to build the systems. The *psfgen* VMD plug-in was utilized to build systems containing protein and ligand structures solvated in a water box extending at least 15 Å beyond every protein atom with the VMD *solvate* plug-in. All systems were neutralized by randomly placing Na^+^ and Cl^−^ ions in the water box using the *autoionize* VMD plug-in. The size of the resulting systems was approximately 60 × 60 × 60 Å^3^ and the number of atoms was roughly 23 thousand atoms. The integration time step was set to 2 fs. A cutoff of 12 Å for van der Waals interactions was used and the particle-mesh Ewald method was used to compute long-range electrostatic forces. Langevin dynamics was utilized to maintain a constant temperature with the damping coefficient set to 5 ps^−1^. The constant pressure equal to 1 atm was maintained using a hybrid Nosé-Hoover-Langevin piston method with a decay period of 50 fs. In all MD simulations periodic boundary conditions in all directions were used. Initially, systems were minimized with conjugate gradient and line search algorithm implemented in NAMD for 5,000 steps without any constrains on the protein and water molecules. Subsequently, the temperature of the system was raised from 0 K to 300 K in 25 K steps (25 ps each step), for the total 300 ps time steps. Finally, the system was equilibrated for 700 ps. The production simulations were run for 20 ns and were used to collect atom fluctuations of the protein and ligands. For RMS deviation and fluctuations analysis the backbone Cα atoms were reoriented relative to the first frame in the production trajectory to remove translational and rotational modes.

Structures were superposed and analysed using the CCP4 suite of programs. All of the structural figures presented were generated using PyMOL (Version 1.8 Schrödinger, LLC).

## Additional Information

**How to cite this article**: Abdullah, S. U. *et al.* Ligand binding to an Allergenic Lipid Transfer Protein Enhances Conformational Flexibility resulting in an Increase in Susceptibility to Gastroduodenal Proteolysis. *Sci. Rep.* **6**, 30279; doi: 10.1038/srep30279 (2016).

## Supplementary Material

Supplementary Information

## Figures and Tables

**Table 1:** Comparative ligand binding characteristics of peach and wheat LTPs at pH 7.5 and 2.5.

Ligand	Buffer	*K*_d_ (μM)		IC_50_ (μM)		*K*_i_ (μM)		ΔG (Kcal/mol)	
		Wheat	Peach	Wheat	Peach	Wheat	Peach	Wheat	Peach
CPA*	50 mM phosphate, pH 7.5	4.5 ± 0.2	1 ± 0.02	—	—	—	—	—	—
CPA*	150 mM NaCl, pH 2.5	1.2 ± 0.1	0.6 ± 0.1	—	—	—	—	—	—
Palmitic acid	50 mM phosphate, pH 7.5	ND^Ψ^	ND	5.2 ± 0.1	1.4 ± 0.1	3.6 ± 0.1	0.7 ± 0.03	7.41	8.39
Linoleic acid	50 mM phosphate, pH 7.5	ND	ND	7.6 ± 0.03	1.6 ± 0.1	5.3 ± 0.02	0.8 ± 0.04	7.19	8.29
Phosphatidyl choline	50 mM phosphate, pH 7.5	ND	ND	6.7 ± 0.1	2.2 ± 0.03	4.6 ± 0.1	1.1 ± 0.02	7.27	8.13
Phosphatidyl choline	150 mM NaCl, pH 2.5	ND	ND	5.3 ± 0.1	1.8 ± 0.1	2.8 ± 0.1	1.0 ± 0.1	7.55	8.17
Phosphatidyl choline (vesicular)	50 mM phosphate, pH 7.5	ND	ND	5.6 ± 0.1	1.5 ± 0.1	3.9 ± 0.1	0.7 ± 0.04	7.37	8.35
Phosphatidyl choline (vesicular)	150 mM NaCl, pH 2.5	ND	ND	6.8 ± 0.2	1.5 ± 0.1	3.6 ± 0.1	0.8 ± 0.1	7.41	8.29

For the remaining ligands, IC_50_. and *K*_i_ values were determined by competitive displacement with CPA.

ΔG was calculated using ΔG = -RTln(*K*_i_).

^Ψ^ND-*K*_d_ not determined; CPA not displaced even at a 3:1 ligand:LTP stoichiometric ratio.

^*^K_d_ values for CPA were determined directly by titration.

**Table 2:** Peptide profiling of 120 min duodenal digests of wheat LTP alone and in the presence of 0.26 mM linoleic acid determined by MALDI-ToF mass spectrometry.

LTP fragments	m/z		Normalised relative intensity	
	Observed	Calculated	Wheat LTP alone	Wheat LTP + linoleic acid
**Intact protein (residues 1–90)**	10063.22	10063.18	518	705
1–34	3474.74	3474.93	160	292
**1–39**	4310.6	4309.77	2546	3290
**1–56**	6243.58	6242.91	537	1047
1–67	7477.3	7477.18	214	290
**1–79**	8803.44	8803.75	270	2300
17–39*	2436.75	2436.65	805	1290
17–56	4370.01	4369.78	533	ND
17–61	4904.39	4904.39	90	90
40–56	1951.23	1951.15	292	ND
40–67	3185.43	3185.43	248	340
**40–79**	4512.11	4512.0	128	656
57–67*	1252.34	1252.29	450	ND
57–89	3739.16	3739.16	110	130
68–79*	1344.71	1344.58	334	ND
80–90*	1277.74	1277.45	1550	1200
Unassigned	3514.0	—	2833	1668
Unassigned	1098.54	—	1320	ND
Unassigned	2679.24	—	1373	1927
Unassigned	4334.4	—	1027	1070
Unassigned	5032.13	—	435	ND
Unassigned	5379.31	—	479	ND
Unassigned	5794.72	—	1004	ND
Unassigned	8673.0	—	312	400

Peptide assignments were derived from comparison of experimentally derived masses with those obtained through *in silico* digestion of wheat LTP (Uniprot ID P24296, Figure S2) with trypsin and chymotrypsin. Peptide assignments corresponding to the most intense mass events are in bold. Asterisks denote peptides that were confirmed by LC-MS/MS. ND- not detected.

**Table 3:** Solvent accessible surface area of Tyr79 of wheat, peach and barley LTPs.

LTP	Unliganded (Å 2 )	1 ligand (Å 2 ) bound	2 ligands (Å 2 ) bound
Wheat	5 (1GH1)	38 (1CZ2)	83 (1BWO)
Peach	22 (2ALG mol B)ϑ	—	52 (2ALG mol A)

Surface area values are averaged over the fifteen, twelve and four ensemble models of the 1GH1, 1CZ2 and 1LIP NMR structures, respectively. For the crystal structures, values are averaged over the two molecules present in each of the asymmetric units of 3GSH and 1BWO (the asymmetric unit of 1MID contains a single LTP molecule). PDB accession codes are in parenthesis.

^ϑ^Molecule B present in the asymmetric unit of the peach LTP crystal structure 2ALG was observed to bind a single lauric acid molecule in an unconventional manner, with the ligand only partially occupying the cavity (Fig. 4D). Therefore, for the purposes of this study, the LTP molecule is regarded as “unliganded”.

## References

[b1] Kader J. C., Julienne M. & Vergnolle C. Purification and characterization of a spinach-leaf protein capable of transferring phospholipids from liposomes to mitochondria or chloroplasts. European journal of biochemistry/FEBS 139, 411–416 (1984). 10.1111/j.1432-1033.1984.tb08020.x 6698022

[b2] Chae K., Kieslich C. A., Morikis D., Kim S. C. & Lord E. M. A gain-of-function mutation of Arabidopsis lipid transfer protein 5 disturbs pollen tube tip growth and fertilization. The Plant cell 21, 3902–3914, doi: 10.1105/tpc.109.070854 (2009). 20044438 PMC2814499

[b3] Pastorello E. A. *et al.* Complete amino acid sequence determination of the major allergen of peach (Prunus persica) Pru p 1. Biological chemistry 380, 1315–1320, doi: 10.1515/BC.1999.167 (1999). 10614824

[b4] Sanchez-Monge R., Lombardero M., Garcia-Selles F. J., Barber D. & Salcedo G. Lipid-transfer proteins are relevant allergens in fruit allergy. The Journal of allergy and clinical immunology 103, 514–519 (1999). 10069888 10.1016/s0091-6749(99)70479-3

[b5] Fernandez-Rivas M. *et al.* Apple allergy across Europe: how allergen sensitization profiles determine the clinical expression of allergies to plant foods. The Journal of allergy and clinical immunology 118, 481–488, doi: 10.1016/j.jaci.2006.05.012 (2006). 16890775

[b6] Hoffmann-Sommergruber K. & Mills E. N. Food allergen protein families and their structural characteristics and application in component-resolved diagnosis: new data from the EuroPrevall project. Analytical and bioanalytical chemistry 395, 25–35, doi: 10.1007/s00216-009-2953-z (2009). 19639305

[b7] Flinterman A. E., Akkerdaas J. H., Knulst A. C., van Ree R. & Pasmans S. G. Hazelnut allergy: from pollen-associated mild allergy to severe anaphylactic reactions. Current opinion in allergy and clinical immunology 8, 261–265, doi: 10.1097/ACI.0b013e3282ffb145 (2008). 18560303

[b8] Palacin A. *et al.* Wheat lipid transfer protein is a major allergen associated with baker’s asthma. The Journal of allergy and clinical immunology 120, 1132–1138, doi: 10.1016/j.jaci.2007.07.008 (2007). 17716720

[b9] Pastorello E. A. *et al.* Wheat IgE-mediated food allergy in European patients: alpha-amylase inhibitors, lipid transfer proteins and low-molecular-weight glutenins. Allergenic molecules recognized by double-blind, placebo-controlled food challenge. International archives of allergy and immunology 144, 10–22, doi: 10.1159/000102609 (2007). 17496422

[b10] Mills E. N., Jenkins J. A., Alcocer M. J. & Shewry P. R. Structural, biological, and evolutionary relationships of plant food allergens sensitizing via the gastrointestinal tract. Critical reviews in food science and nutrition 44, 379–407, doi: 10.1080/10408690490489224 (2004). 15540651

[b11] Charvolin D., Douliez J. P., Marion D., Cohen-Addad C. & Pebay-Peyroula E. The crystal structure of a wheat nonspecific lipid transfer protein (ns-LTP1) complexed with two molecules of phospholipid at 2.1 A resolution. European journal of biochemistry/FEBS 264, 562–568 (1999). 10.1046/j.1432-1327.1999.00667.x 10491104

[b12] Lerche M. H. & Poulsen F. M. Solution structure of barley lipid transfer protein complexed with palmitate. Two different binding modes of palmitate in the homologous maize and barley nonspecific lipid transfer proteins. Protein science: a publication of the Protein Society 7, 2490–2498, doi: 10.1002/pro.5560071202 (1998). 9865943 PMC2143888

[b13] Pasquato N. *et al.* Crystal structure of peach Pru p 3, the prototypic member of the family of plant non-specific lipid transfer protein pan-allergens. Journal of molecular biology 356, 684–694, doi: 10.1016/j.jmb.2005.11.063 (2006). 16388823

[b14] Bakan B. *et al.* Specific adduction of plant lipid transfer protein by an allene oxide generated by 9-lipoxygenase and allene oxide synthase. The Journal of biological chemistry 281, 38981–38988, doi: 10.1074/jbc.M608580200 (2006). 17046828

[b15] Lindorff-Larsen K. & Winther J. R. Surprisingly high stability of barley lipid transfer protein, LTP1, towards denaturant, heat and proteases. FEBS letters 488, 145–148 (2001). 11163761 10.1016/s0014-5793(00)02424-8

[b16] Sy D., Le Gravier Y., Goodfellow J. & Vovelle F. Protein stability and plasticity of the hydrophobic cavity in wheat ns-LTP. Journal of biomolecular structure & dynamics 21, 15–29, doi: 10.1080/07391102.2003.10506902 (2003). 12854956

[b17] Sodano P. *et al.* 1H NMR and fluorescence studies of the complexation of DMPG by wheat non-specific lipid transfer protein. Global fold of the complex. FEBS letters 416, 130–134 (1997). 9369197 10.1016/s0014-5793(97)01185-x

[b18] Wijesinha-Bettoni R. *et al.* Post-translational modification of barley LTP1b: the lipid adduct lies in the hydrophobic cavity and alters the protein dynamics. FEBS letters 581, 4557–4561, doi: 10.1016/j.febslet.2007.08.041 (2007). 17854802

[b19] Breiteneder H. & Mills E. N. Molecular properties of food allergens. The Journal of allergy and clinical immunology 115, 14–23; quiz 24, doi: 10.1016/j.jaci.2004.10.022 (2005). 15637541

[b20] (EFSA), E. F. S. A. Scientific Opinion on the assessment of allergenicity of GM plants and microorganisms and derived food and feed-EFSA Panel on Genetically Modified Organisms (GMO Panel). EFSA Journal 8, 168, doi: 10.2903/j.efsa.2010.1700 (2010).

[b21] Wijesinha-Bettoni R. *et al.* The structural characteristics of nonspecific lipid transfer proteins explain their resistance to gastroduodenal proteolysis. Biochemistry 49, 2130–2139, doi: 10.1021/bi901939z (2010). 20121231

[b22] Cooper D. J., Husband F. A., Mills E. N. & Wilde P. J. Role of beer lipid-binding proteins in preventing lipid destabilization of foam. Journal of agricultural and food chemistry 50, 7645–7650 (2002). 12475284 10.1021/jf0203996

[b23] Izzo R. *et al.* Lipid Evolution during Development and Ripening of Peach Fruits. Phytochemistry 39, 1329–1334, doi: 10.1016/0031-9422(95)00189-E (1995).

[b24] Morrison W. R. Wheat Lipid-Composition. Cereal Chem 55, 548–558 (1978).

[b25] Bengmark S. Immunonutrition: role of biosurfactants, fiber, and probiotic bacteria. Nutrition 14, 585–594 (1998). 9684261 10.1016/s0899-9007(98)00030-6

[b26] Douliez J. P., Michon T. & Marion D. Steady-state tyrosine fluorescence to study the lipid-binding properties of a wheat non-specific lipid-transfer protein (nsLTP1). Biochimica et biophysica acta 1467, 65–72 (2000). 11004474

[b27] Subirade M., Salesse C., Marion D. & Pezolet M. Interaction of a nonspecific wheat lipid transfer protein with phospholipid monolayers imaged by fluorescence microscopy and studied by infrared spectroscopy. Biophysical journal 69, 974–988, doi: 10.1016/S0006-3495(95)79971-4 (1995). 8519997 PMC1236326

[b28] Yapoudjian S. *et al.* Binding of Thermomyces (Humicola) lanuginosa lipase to the mixed micelles of cis-parinaric acid/NaTDC. European journal of biochemistry/FEBS 269, 1613–1621 (2002). 10.1046/j.1432-1327.2002.02786.x 11895431

[b29] Lei L. *et al.* A nodule-specific lipid transfer protein AsE246 participates in transport of plant-synthesized lipids to symbiosome membrane and is essential for nodule organogenesis in Chinese milk vetch. Plant physiology 164, 1045–1058, doi: 10.1104/pp.113.232637 (2014). 24367021 PMC3912078

[b30] Asero R. *et al.* Lipid transfer protein: a pan-allergen in plant-derived foods that is highly resistant to pepsin digestion. International archives of allergy and immunology 124, 67–69, doi: 53671 (2001). 11306929 10.1159/000053671

[b31] Hubbard S. J., Beynon R. J. & Thornton J. M. Assessment of conformational parameters as predictors of limited proteolytic sites in native protein structures. Protein engineering 11, 349–359 (1998). 9681867 10.1093/protein/11.5.349

[b32] Yeats T. H. & Rose J. K. The biochemistry and biology of extracellular plant lipid-transfer proteins (LTPs). Protein science: a publication of the Protein Society 17, 191–198, doi: 10.1110/ps.073300108 (2008). 18096636 PMC2222726

[b33] De Libero G. & Mori L. Recognition of lipid antigens by T cells. Nat Rev Immunol 5, 485–496, doi: 10.1038/nri1631 (2005). 15928680

[b34] Bublin M., Eiwegger T. & Breiteneder H. Do lipids influence the allergic sensitization process? The Journal of allergy and clinical immunology 134, 521–529, doi: 10.1016/j.jaci.2014.04.015 (2014). 24880633 PMC4151997

[b35] Bossios A. *et al.* Effect of simulated gastro-duodenal digestion on the allergenic reactivity of beta-lactoglobulin. Clinical and translational allergy 1, 6, doi: 10.1186/2045-7022-1-6 (2011). 22410304 PMC3339358

[b36] Moreno F. J., Mackie A. R. & Mills E. N. Phospholipid interactions protect the milk allergen alpha-lactalbumin from proteolysis during *in vitro* digestion. Journal of agricultural and food chemistry 53, 9810–9816, doi: 10.1021/jf0515227 (2005). 16332136

[b37] Petersen A. *et al.* Roasting and lipid binding provide allergenic and proteolytic stability to the peanut allergen Ara h 8. Biological chemistry 395, 239–250, doi: 10.1515/hsz-2013-0206 (2014). 24057594

[b38] Berecz B. *et al.* Stability of sunflower 2S albumins and LTP to physiologically relevant *in vitro* gastrointestinal digestion. Food chemistry 138, 2374–2381, doi: 10.1016/j.foodchem.2012.12.034 (2013). 23497898

[b39] Vassilopoulou E. *et al.* Effect of *in vitro* gastric and duodenal digestion on the allergenicity of grape lipid transfer protein. The Journal of allergy and clinical immunology 118, 473–480, doi: 10.1016/j.jaci.2006.04.057 (2006). 16890774

[b40] Bakan B. *et al.* The crystal structure of oxylipin-conjugated barley LTP1 highlights the unique plasticity of the hydrophobic cavity of these plant lipid-binding proteins. Biochemical and biophysical research communications 390, 780–785, doi: 10.1016/j.bbrc.2009.10.049 (2009). 19836358

[b41] Nielsen S. U. & Spener F. Fatty acid-binding protein from rat heart is phosphorylated on Tyr19 in response to insulin stimulation. Journal of lipid research 34, 1355–1366 (1993). 7691977

[b42] de Jong A. J., Kloppenburg M., Toes R. E. & Ioan-Facsinay A. Fatty acids, lipid mediators, and T-cell function. Frontiers in immunology 5, 483, doi: 10.3389/fimmu.2014.00483 (2014). 25352844 PMC4195378

[b43] Mirotti L. *et al.* Lipids are required for the development of Brazil nut allergy: the role of mouse and human iNKT cells. Allergy 68, 74–83, doi: 10.1111/all.12057 (2013). 23137012

[b44] Mills E. N., Sancho A. I., Rigby N. M., Jenkins J. A. & Mackie A. R. Impact of food processing on the structural and allergenic properties of food allergens. Molecular nutrition & food research 53, 963–969, doi: 10.1002/mnfr.200800236 (2009). 19603402

[b45] Verhoeckx K. C. *et al.* Food processing and allergenicity. Food Chem Toxicol 80, 223–240, doi: 10.1016/j.fct.2015.03.005 (2015). 25778347

[b46] Bogh K. L. & Madsen C. B. Food allergens: Is There a Correlation between Stability to Digestion and Allergenicity? Critical reviews in food science and nutrition 0, doi: 10.1080/10408398.2013.779569 (2015). 25607526

[b47] Jegou S., Douliez J. P., Molle D., Boivin P. & Marion D. Purification and structural characterization of LTP1 polypeptides from beer. Journal of agricultural and food chemistry 48, 5023–5029 (2000). 11052772 10.1021/jf000075m

[b48] Gaier S. *et al.* Purification and structural stability of the peach allergens Pru p 1 and Pru p 3. Molecular nutrition & food research 52 Suppl 2, S220–S229, doi: 10.1002/mnfr.200700274 (2008). 18384093

[b49] Hofliger M. M., Castejon G. L., Kiess W. & Beck Sickinger A. G. Novel cell line selectively expressing neuropeptide Y-Y2 receptors. Journal of receptor and signal transduction research 23, 351–360, doi: 10.1081/RRS-120026974 (2003). 14753296

[b50] Kane C. D., Coe N. R., Vanlandingham B., Krieg P. & Bernlohr D. A. Expression, purification, and ligand-binding analysis of recombinant keratinocyte lipid-binding protein (MAL-1), an intracellular lipid-binding found overexpressed in neoplastic skin cells. Biochemistry 35, 2894–2900, doi: 10.1021/bi952476e (1996). 8608126

[b51] Cheng Y. & Prusoff W. H. Relationship between the inhibition constant (K1) and the concentration of inhibitor which causes 50 per cent inhibition (I50) of an enzymatic reaction. Biochemical pharmacology 22, 3099–3108 (1973). 4202581 10.1016/0006-2952(73)90196-2

[b52] Minekus M. *et al.* A standardised static *in vitro* digestion method suitable for food-an international consensus. Food Funct 5, 1113–1124, doi: 10.1039/c3fo60702j (2014). 24803111

[b53] Moreno F. J., Mellon F. A., Wickham M. S., Bottrill A. R. & Mills E. N. Stability of the major allergen Brazil nut 2S albumin (Ber e 1) to physiologically relevant *in vitro* gastrointestinal digestion. The FEBS journal 272, 341–352, doi: 10.1111/j.1742-4658.2004.04472.x (2005). 15654873

[b54] Palacin A. *et al.* Recombinant lipid transfer protein Tri a 14: a novel heat and proteolytic resistant tool for the diagnosis of baker’s asthma. Clin Exp Allergy 39, 1267–1276, doi: 10.1111/j.1365-2222.2009.03280.x (2009). 19486028

[b55] Strohalm M., Hassman M., Kosata B. & Kodicek M. mMass data miner: an open source alternative for mass spectrometric data analysis. Rapid communications in mass spectrometry: RCM 22, 905–908, doi: 10.1002/rcm.3444 (2008). 18293430

[b56] Sievers F. *et al.* Fast, scalable generation of high-quality protein multiple sequence alignments using Clustal Omega. Mol Syst Biol 7, 539, doi: 10.1038/msb.2011.75 (2011). 21988835 PMC3261699

[b57] Gouet P., Courcelle E., Stuart D. I. & Metoz F. ESPript: analysis of multiple sequence alignments in PostScript. Bioinformatics 15, 305–308 (1999). 10320398 10.1093/bioinformatics/15.4.305

[b58] Gincel E. *et al.* Three-dimensional structure in solution of a wheat lipid-transfer protein from multidimensional 1H-NMR data. A new folding for lipid carriers. European journal of biochemistry/FEBS 226, 413–422 (1994). 10.1111/j.1432-1033.1994.tb20066.x 8001559

[b59] Tassin-Moindrot S., Caille A., Douliez J. P., Marion D. & Vovelle F. The wide binding properties of a wheat nonspecific lipid transfer protein. Solution structure of a complex with prostaglandin B2. European journal of biochemistry/FEBS 267, 1117–1124 (2000). 10.1046/j.1432-1327.2000.01109.x 10672021

[b60] Kelley L. A. & Sutcliffe M. J. OLDERADO: on-line database of ensemble representatives and domains. On Line Database of Ensemble Representatives And DOmains. Protein science: a publication of the Protein Society 6, 2628–2630, doi: 10.1002/pro.5560061215 (1997). 9416612 PMC2143626

[b61] Jo S., Kim T., Iyer V. G. & Im W. CHARMM-GUI: a web-based graphical user interface for CHARMM. J Comput Chem 29, 1859–1865, doi: 10.1002/jcc.20945 (2008). 18351591

[b62] Vanommeslaeghe K. & MacKerell A. D. Jr. Automation of the CHARMM General Force Field (CGenFF) I: bond perception and atom typing. J Chem Inf Model 52, 3144–3154, doi: 10.1021/ci300363c (2012). 23146088 PMC3528824

[b63] Humphrey W., Dalke A. & Schulten K. VMD: Visual molecular dynamics. J Mol Graph Model 14, 33–38, doi: 10.1016/0263-7855(96)00018-5 (1996). 8744570

[b64] Phillips J. C. *et al.* Scalable molecular dynamics with NAMD. J Comput Chem 26, 1781–1802, doi: 10.1002/Jcc.20289 (2005). 16222654 PMC2486339

