# Supplementary material for: Ligand binding to an Allergenic Lipid Transfer Protein Enhances Conformational Flexibility resulting in an Increase in Susceptibility to Gastroduodenal Proteolysis
Source: Sci Rep. 2016 Jul 26;6:30279. doi: 10.1038/srep30279 (PMC4960534; doi:10.1038/srep30279)

SUPPLEMENTARY INFORMATION

Ligand binding to an Allergenic Lipid Transfer Protein Enhances Conformational Flexibility resulting in an Increase in Susceptibility to Gastroduodenal Proteolysis

Syed Umer Abdullah[1†], Yuri Alexeev[1,2†], Philip E. Johnson [1,3,4†], Neil M. Rigby[1], Alan R. Mackie[1], Balvinder Dhaliwal[4]* and E.N. Clare Mills [1, 4]

[1] Institute of Food Research, Norwich Research Park, Colney, NR4 7UA, U.K.

[2] Current address: Argonne Leadership Computing Facility, Argonne National Laboratory, Argonne, Illinois 60439, U.S.A.

[3] Current address: Department of Food Science & Technology, Food Allergy Research & Resource Program, University of Nebraska-Lincoln, 266 Food Innovation Center, 1901 North 21st Street Lincoln, NE 68588-6205.

[4] Centre for Respiratory Medicine and Allergy, Division of Infection, Immunity & Respiratory Medicine, School of Biological Sciences, Faculty of Biology, Medicine and Health, Manchester Academic Health Sciences Centre and Manchester Institute of Biotechnology, University of Manchester, 131 Princess Street, Manchester, M1 7DN, U.K.

* Corresponding author: -

Dr. Balvinder Dhaliwal: Phone: +44 (0)161 306 4214, E-mail: balvinder.dhaliwal@manchester.ac.uk

† These authors contributed equally to the data in this manuscript.

Supplementary table

Table S1. RMS deviations (RMSD) between unliganded and liganded wheat LTP structures. RMSD values for all Cα atom pairs (residues 1 to 90) are shown in bold, and RMSD values for Cα atom pairs of the unstructured region (residues 74 to 90) are shown in italic.

**RMSD (Å)**	Without Ligand	1 ligand	2 ligands
Without ligand	-	*4.0*	*3.4*
1 ligand	**1.8**	-	*3.1*
2 ligands	**1.5**	**1.7**	-

Supplementary figure legends

Figure S1. Wheat LTP is resistant to gastric digestion. (A, B) SDS PAGE analysis of digestion under reducing conditions in the absence (A) or presence (B) of 0.26mM linoleic acid (LA); lane 0 shows intact wheat LTP. (C, D) MALDI-ToF MS spectra of the gastric digests of wheat LTP after 60 min in the absence (C) or presence (D) of 0.26 mM linoleic acid. (E) Densitometric analysis of SDS PAGE shown in (A) and (B).

Figure S2. Peach LTP is resistant to gastric digestion. (A, B) SDS PAGE analysis of digestion under reducing conditions in the absence (A) or presence (B) of 0.26mM linoleic acid (LA); lane 0 shows intact peach LTP. (C, D) MALDI-ToF MS spectra of the gastric digests of peach LTP after 60 min in the absence (C) or presence (D) of 0.26 mM linoleic acid. (E) Densitometric analysis of SDS PAGE shown in (A) and (B).

Figure S3. Duodenal digestion of peach LTP. (A, B) SDS PAGE analysis of digestion under reducing conditions at various time points in the absence (A) or presence (B) of 0.26mM linoleic acid (LA); lane R is a reference showing peach LTP following the duodenal digestion procedure but in the absence of trypsin and chymotrypsin. Bands associated with a stable peptide fragment (residues 1 to 79) are highlighted. (C) MALDI-ToF MS spectra of the duodenal digests of peach LTP after 120 min in the absence (red spectra) or presence (blue) of 0.26 mM linoleic acid. (D) Densitometric analysis of SDS PAGE shown in (A) and (B).

Figure S4. Sequence alignment of peach and wheat LTPs. Alignment of peach (Uniprot ID Q9LED1) and wheat (P24296) LTP showing theoretical chymotrypsin (green arrows), trypsin (orange arrows) and observed (black circles) cleavage sites. Strictly conserved residues have a red background and those well-conserved are indicated by red lettering.

Supplementary figures

Figure S1

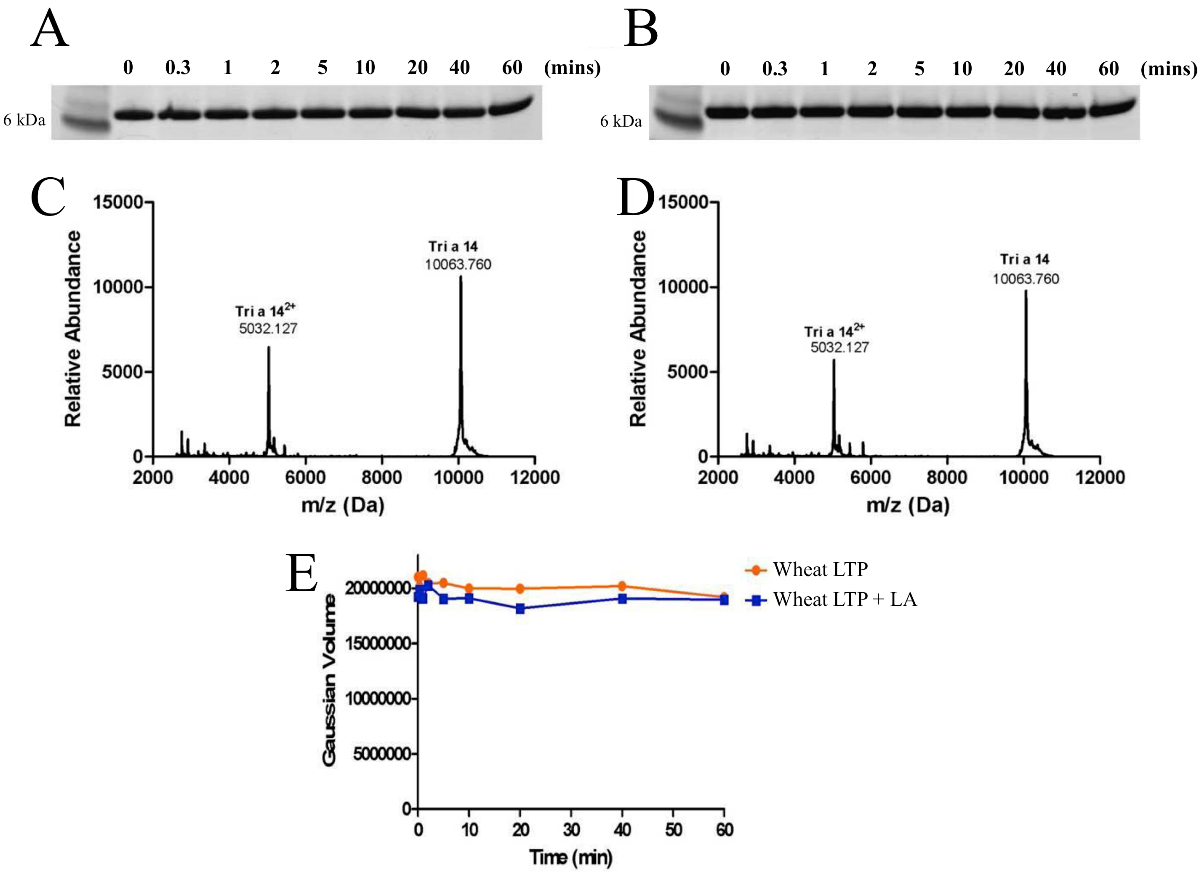

Figure S2

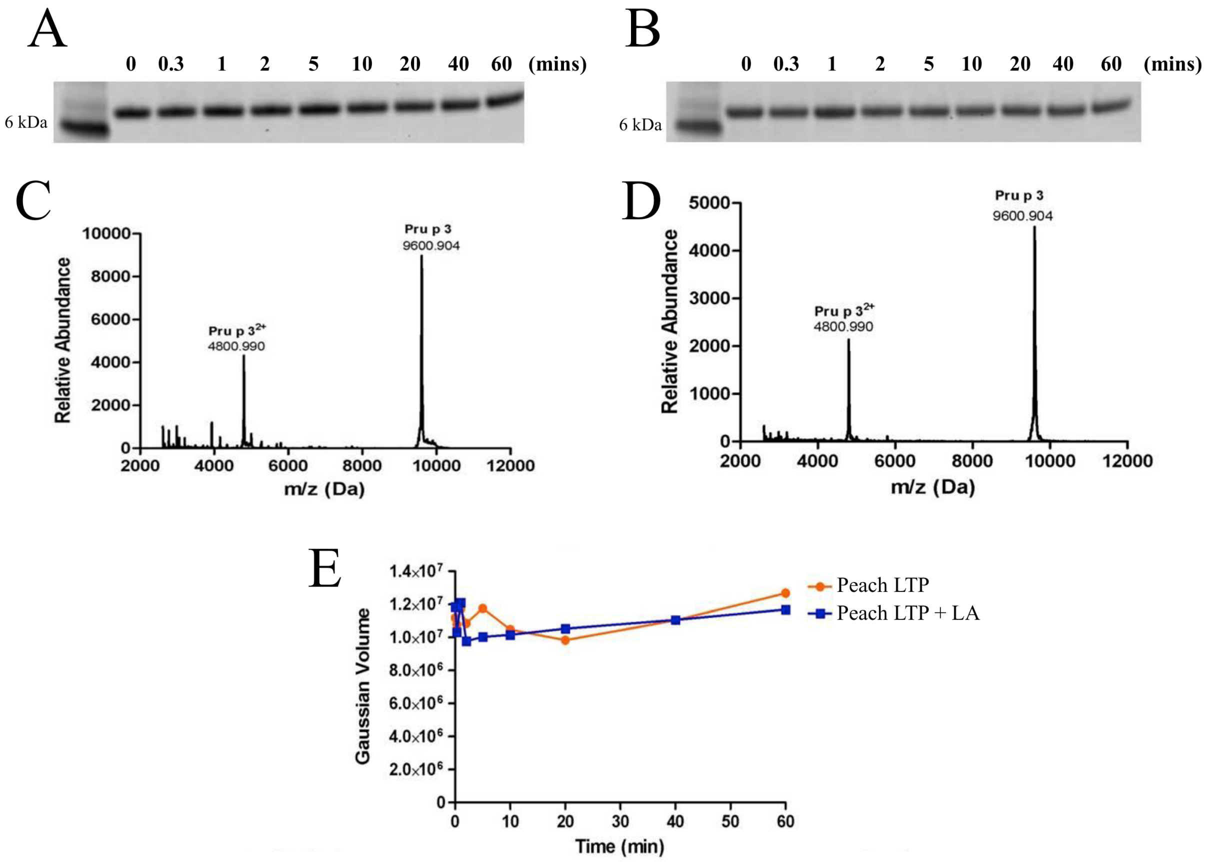
A
0 0.3 1 2 5 10 20 40 60 (mins)
6 kDa
B
0 0.3 1 2 5 10 20 40 60 (mins)
6 kDa
C
Relative Abundance
m/z (Da)
Pru p 3
9600.904
Pru p 3^{2+}
4800.990
D
Relative Abundance
m/z (Da)
Pru p 3
9600.904
Pru p 3^{2+}
4800.990
E
Gaussian Volume
Time (min)
Peach LTP
Peach LTP + LA

Figure S3

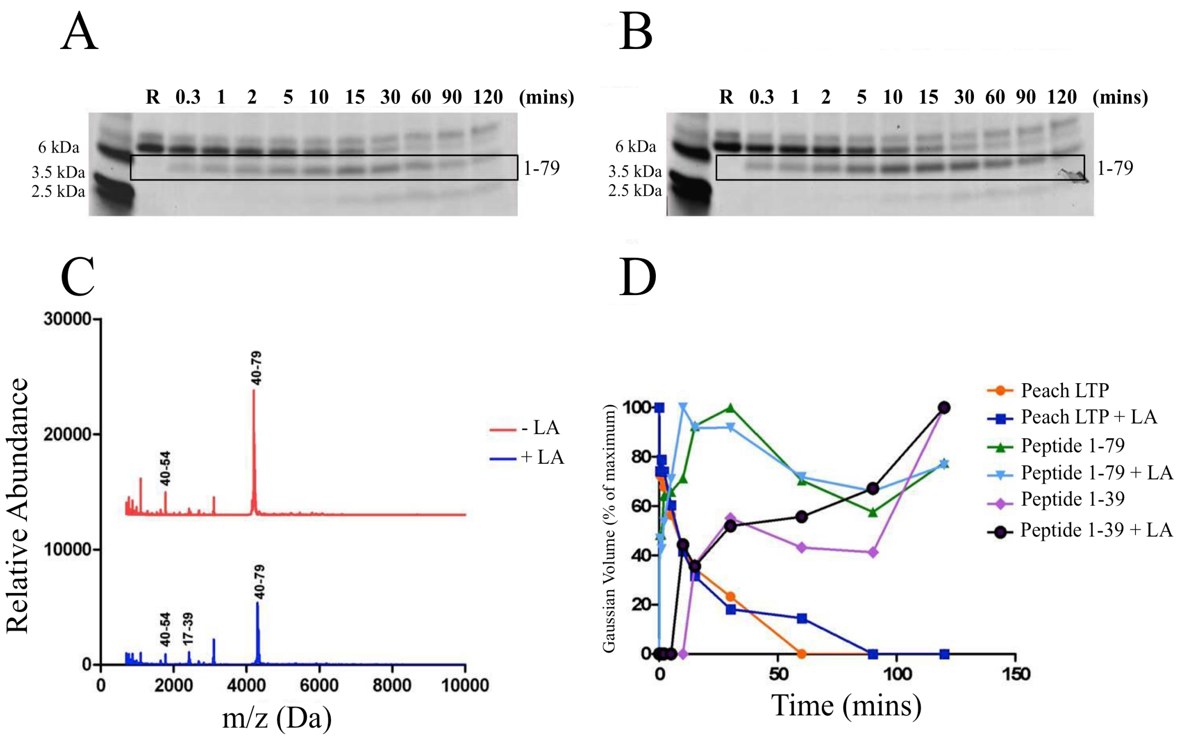

Figure S4

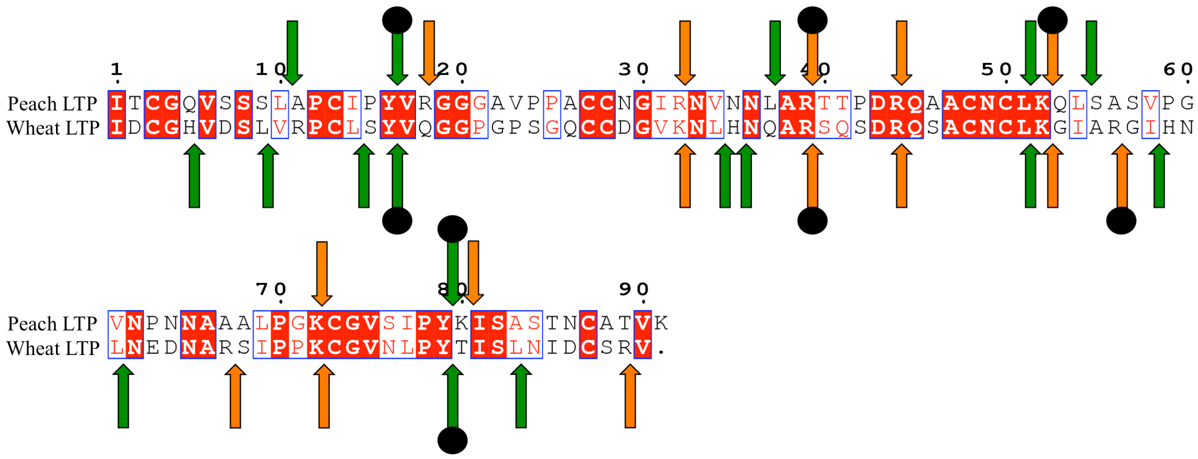

Supplement: Supplementary Information [file srep30279-s1.pdf]
